# Associations between food group intakes and circulating insulin-like growth factor-I in the UK Biobank: a cross-sectional analysis

**DOI:** 10.1007/s00394-022-02954-4

**Published:** 2022-07-30

**Authors:** Cody Z. Watling, Rebecca K. Kelly, Tammy Y. N. Tong, Carmen Piernas, Eleanor L. Watts, Sandar Tin Tin, Anika Knuppel, Julie A. Schmidt, Ruth C. Travis, Timothy J. Key, Aurora Perez-Cornago

**Affiliations:** 1 Cancer Epidemiology Unit, Nuffield Department of Population Health, University of Oxford, Oxford, OX3 7LF UK; 2 Nuffield Department of Primary Care Health Sciences, University of Oxford, Oxford, UK; 3 Department of Clinical Epidemiology, Department of Clinical Medicine, Aarhus University and Aarhus University Hospital, Aarhus, Denmark; 4 MRC Unit for Lifelong Health and Ageing at UCL, Institute of Cardiovascular Science, University College London, London, UK; 5 Department of Biochemistry and Molecular Biology II, Center for Biomedical Research (CIBM), University of Granada, Granada, Spain

**Keywords:** Food, Diet, IGF-I, Somatomedin C, Poultry, Fish

## Abstract

**Purpose:**

Circulating insulin-like growth factor-I (IGF-I) concentrations have been positively associated with risk of several common cancers and inversely associated with risk of bone fractures. Intakes of some foods have been associated with increased circulating IGF-I concentrations; however, evidence remains inconclusive. Our aim was to assess cross-sectional associations of food group intakes with circulating IGF-I concentrations in the UK Biobank.

**Methods:**

At recruitment, the UK Biobank participants reported their intake of commonly consumed foods. From these questions, intakes of total vegetables, fresh fruit, red meat, processed meat, poultry, oily fish, non-oily fish, and cheese were estimated. Serum IGF-I concentrations were measured in blood samples collected at recruitment. After exclusions, a total of 438,453 participants were included in this study. Multivariable linear regression was used to assess the associations of food group intakes with circulating IGF-I concentrations.

**Results:**

Compared to never consumers, participants who reported consuming oily fish or non-oily fish ≥ 2 times/week had 1.25 nmol/L (95% confidence interval:1.19–1.31) and 1.16 nmol/L (1.08–1.24) higher IGF-I concentrations, respectively. Participants who reported consuming poultry ≥ 2 times/week had 0.87 nmol/L (0.80–0.94) higher IGF-I concentrations than those who reported never consuming poultry. There were no strong associations between other food groups and IGF-I concentrations.

**Conclusions:**

We found positive associations between oily and non-oily fish intake and circulating IGF-I concentrations. A weaker positive association of IGF-I with poultry intake was also observed. Further research is needed to understand the mechanisms which might explain these associations.

**Supplementary Information:**

The online version contains supplementary material available at 10.1007/s00394-022-02954-4.

## Introduction

Insulin-like growth factor-I (IGF-I) is a hormone, primarily produced in the liver, which stimulates cell growth and proliferation [1]. In prospective and genetic studies, higher circulating IGF-I concentrations have been associated with several health outcomes including higher risks of breast, colorectal, and prostate cancer [2–6], and higher bone mineral density and lower risk of bone fracture [7, 8].

While there is substantial evidence assessing dietary factors associated with circulating IGF-I concentrations [9–13], to date the evidence seems consistent only for dairy products. Previous cross-sectional studies [9–12] and randomised controlled trials [14–17], have reported some evidence for a positive association between intake of dairy products and IGF-I concentrations, which has been proposed to be due to the protein content in dairy products [9, 18]. However, intake of dairy products and IGF-I concentrations may vary by dairy sources [18, 19] with our previous work assessing nutrient intakes suggesting that intake of protein from milk and yogurt, but not cheese, is positively associated with IGF-I concentrations [18]. Moreover, it is not well understood whether or how intakes of other protein-rich foods, such as red meat, poultry and fish, and other food groups, such as fruits and vegetables, relate to circulating IGF-I concentrations, with previous studies being limited by relatively small sample sizes [9–12].

The UK Biobank cohort study collected dietary information and measured serum IGF-I concentrations in blood collected at baseline from nearly 500,000 participants in the United Kingdom. Using this resource, we conducted a cross-sectional analysis to assess the associations of selected food groups with circulating IGF-I concentrations.

## Materials and methods

### Study design and participants

Eligible individuals were identified for invitation to participate in the UK Biobank (total 9.2 million individuals) using the National Health Service patient registers. In total 503,317 individuals aged 37–73 years consented to enrol (5.5% response rate) from 2006 to 2010 [20]. At recruitment, participants attended an assessment centre and provided informed consent and detailed information about diet, lifestyle, sociodemographic, and reproductive factors via a touchscreen questionnaire. Anthropometric measurements were made using standardized procedures [21], and blood samples were taken [22]. Ethical approval was obtained from the Northwest Multi-Centre Research Ethics Committee (reference number 21/NW/0157). A full description of the study assessment, protocol, and ethical approval can be found on the UK Biobank website [23].

### Exclusions

At the time of this analysis, a total of 824 participants had withdrawn their informed consent from the study and were excluded. Participants were also excluded if they had a prevalent cancer at recruitment recorded by a cancer registry (excluding non-melanoma skin cancer; *N* = 27,174), were taking medications which may alter IGF-I concentrations, such as growth hormone (*N* = 4077; Supplementary Table S1), or did not have a measured value for IGF-I at recruitment (*N* = 32,789). In total, a maximum of 438,453 participants were included in this analysis (Supplementary Figure S1 shows flow chart of exclusions).

### Dietary assessment

At recruitment, participants were asked to report how frequently they consumed 14 common foods on a weekly or daily basis over the past 12 months as part of a short touchscreen questionnaire [24]. For the current study, for each of the following foods and food groups, participants were categorised into four groups based on their reported frequency of intake: vegetables (raw and cooked), fresh fruit, red meat (unprocessed beef, pork, and lamb/mutton), processed meat (e.g., bacon, ham, sausages), poultry, oily fish (e.g., sardines, salmon, mackerel, herring), non-oily fish (e.g., cod, tinned tuna, haddock), and cheese. Cut-offs for categories were chosen based on the data distribution of intakes for each food group. Further information on calculation of serving sizes and categorisation has been reported elsewhere [25, 26]. Briefly, for consumption of vegetables and fresh fruit, participants were asked to enter the number of heaped tablespoons (for both cooked vegetables and salad/raw vegetables) or pieces of fruit (with examples as “one apple, one banana, 10 grapes” constituting one piece) consumed per day. Participants also had the option to select ‘less than one’, ‘do not know’ or ‘prefer not to answer’ for questions on cooked vegetables, raw vegetables, or fresh fruit intake. For oily fish, non-oily fish, processed meats, poultry, beef, lamb, pork, and cheese, no portion size was given in the question, instead, participants were asked how often each item was consumed with possible choices being: ‘never, ‘less than once a week’, ‘2–4 times a week’, ‘5–6 times a week’, ‘once or more daily’, ‘do not know’, or ‘prefer not to answer’. From these responses, participants were categorised into intakes for each food group based on their reported consumption.

### Laboratory analysis

Non-fasting blood samples were collected from nearly all participants (99.7%) at the recruitment visit and were transported at 4 °C to the central laboratory for cryopreservation and subsequent biochemical measurements. Serum concentrations of IGF-I were measured using the DiaSorin Ltd. LIAISON® XL chemiluminescent immunoassay. The coefficient of variation for circulating IGF-I concentrations at baseline was 26.5%. Details about assay methods and quality control procedures for serum blood measurements are available online [22].

### Repeat assessment

Participants who lived within a 35 km radius were invited to attend a repeat baseline assessment at the UK Biobank Centre in Stockport between August 2012 and June 2013, ~ 4 years after their initial visit. At this repeat baseline assessment, participants had measurements retaken, completed the questionnaire from the recruitment visit, and provided a second blood sample. Further information on the UK Biobank repeat visit can be found on the UK Biobank website [27]. From this follow-up visit, a total of 16,689 participants had a valid IGF-I concentration measured with 15,419 participants having both IGF-I measurements. Pearson correlations between the baseline IGF-I measurement and second measurement for the same individuals were *r* = 0.76 for all, *r* = 0.77 for men, and *r* = 0.74 for women.

### Statistical analysis

Circulating IGF-I concentrations were logarithmically transformed to minimize the impact of outliers. The geometric mean concentrations of IGF-I was obtained within each category of intake of food groups from linear regression models. To determine relative values, geometric means in the other categories were divided by the geometric mean in the lowest category.

In minimally adjusted linear regression models, adjustments were made for sex and age at recruitment (< 45, 45–49, 50–54, 55–59, 60–64, ≥ 65 years). Multivariable linear regression models were further adjusted for region of recruitment (North-West England, North-Eastern England, Yorkshire & the Humber, West Midlands, East Midlands, South-East England, South-West England, London, Wales, and Scotland), body mass index (BMI; < 20, 20–22.49, 22.5–24.9, 25–27.49, 27.5–29.9, 30–32.49, 32.5–34.9, ≥ 35 kg/m^2^, and unknown/missing), height (eight sex-specific categories increasing by 5 cm, and unknown/missing), physical activity (low; 0–9.99, medium; 10–49.99, high; ≥ 50 metabolic equivalent of task (MET)-hours/week, and unknown/missing), Townsend deprivation index (quintiles from most deprived to least deprived, or unknown), education (completion of national exam at 16 years of age, completion of national exam at 17–18 years of age, college or university degree, or unknown/missing), smoking status (never, former, light smoker: < 15 cigarettes/day, medium smoker: 15–29 cigarettes/day, heavy smoker: ≥ 30 cigarettes/day, or missing/unknown), alcohol consumption (non-drinkers, < 1, 1–9.99, 10–19.99, ≥ 20 g/day or unknown/missing), ethnicity (white, mixed race, Indian/Pakistani/Bangladeshi, Chinese/Asian, black/black British, other, or missing/unknown), diabetes (yes, no, unknown), and women-specific covariates: hormone replacement therapy (HRT) use (never, former, current, or unknown), oral contraceptive use (never, former, current, or unknown), and menopausal status (premenopausal, postmenopausal, or unknown). Further information on categorisation and classification of covariates have been described elsewhere [18]. Participants who responded as ‘prefer not to answer’ or ‘do not know’ in the touchscreen questionnaire for specific food groups were excluded from the respective analyses.

### Subgroup and sensitivity analyses

Heterogeneity by sex was assessed using a likelihood ratio test comparing the multivariable model to a model including an interaction term between the food group and sex. Sensitivity analyses were conducted in participants who had IGF-I concentrations measured at the reassessment visit (mean 4.3 years after recruitment).

All analyses were conducted using Stata version 17.0 (Stata Corp LP, College Station, TX) and figures were produced using “Jasper makes plots” package version 2–266 in R 4.1.0 [28]. *P*-values were two sided and, with Bonferroni correction so that *p*-values < 0.00625 (0.05/8 exposures) were considered statistically significant. As a result of the large sample size, most results were statistically significant even after correction for multiple testing. As such, only the largest percentage differences of IGF-I concentrations between highest and lowest categories (~ 5% or greater difference) have been described in the text. All models were visually assessed to make sure residuals were normally distributed using Q-Q plots, and not heteroscedastic using residual-versus-fitted plots. No assumptions for linear regression were deemed to be invalid.

## Results

Table 1 shows participant baseline characteristics by quintiles of IGF-I concentrations. Those who had higher IGF-I concentrations were more likely to be men, to be younger and taller, to have lower BMI, and to report they were never smokers.

**Table 1:** Characteristics of participants in UK Biobank by quintiles of circulating IGF-I concentrations (*N* = 438,453)

	Circulating IGF-I				
	Fifth 1	Fifth 2	Fifth 3	Fifth 4	Fifth 5
Number of participants	87,716	87,686	87,687	87,696	87,668
IGF-I concentration, nmol/L	13.9 (2.2)	18.3 (0.9)	21.3 (0.8)	24.1 (0.9)	29.5 (4.0)
IGF-I concentration at follow-up, nmol/L	15.1 (3.6)	18.5 (3.3)	20.8 (3.4)	23.0 (3.6)	26.9 (5.0)
Sex—Male, *N* (%)	32,843 (37.4%)	38,985 (44.5%)	42,241 (48.2%)	44,267 (50.5%)	43,563 (49.7%)
Age, years	59.1 (7.1)	57.6 (7.6)	56.4 (7.9)	55.2 (8.2)	53.3 (8.4)
Body mass index, kg/m2	28.6 (5.7)	27.5 (4.9)	27.2 (4.5)	27.0 (4.3)	26.7 (4.1)
Height, cm	166.3 (9.0)	167.9 (9.1)	168.9 (9.2)	169.6 (9.3)	170.0 (9.3)
*Physical activity, N (%)*					
Low	27,983 (31.9%)	25,238 (28.8%)	24,743 (28.2%)	24,279 (27.7%)	24,268 (27.7%)
Moderate	39,236 (44.7%)	41,613 (47.5%)	42,547 (48.5%)	43,413 (49.5%)	44,324 (50.6%)
High	16,609 (18.9%)	17,518 (20.0%)	17,427 (19.9%)	17,262 (19.7%)	16,355 (18.7%)
Townsend deprivation index, *N* (%)					
Q1—Most affluent	15,780 (18.0%)	17,546 (20.0%)	17,959 (20.5%)	18,385 (21.0%)	18,457 (21.1%)
Q5—Most deprived	20,242 (23.1%)	17,454 (19.9%)	16,698 (19.0%)	16,273 (18.6%)	16,277 (18.6%)
*Education, N (%)*					
National examination at age 16 years	15,029 (17.1%)	14,887 (17.0%)	14,650 (16.7%)	14,445 (16.5%)	14,337 (16.4%)
National examination at age 17–18 years	4466 (5.1%)	4671 (5.3%)	4750 (5.4%)	4909 (5.6%)	5234 (6.0%)
College or University degree	46,604 (53.1%)	50,778 (57.9%)	53,306 (60.8%)	54,863 (62.6%)	56,767 (64.8%)
*Smoking, N (%)*					
Never	44,447 (50.7%)	46,438 (53.0%)	47,966 (54.7%)	49,401 (56.3%)	51,656 (58.9%)
Previous	32,797 (37.4%)	31,151 (35.5%)	30,139 (34.4%)	28,923 (33.0%)	26,912 (30.7%)
Light smoker: < 15 cigarettes/day	2835 (3.2%)	2652 (3.0%)	2638 (3.0%)	2543 (2.9%)	2687 (3.1%)
Medium smoker: 15–29 cigarettes/day	3619 (4.1%)	3353 (3.8%)	3003 (3.4%)	2910 (3.3%)	2861 (3.3%)
Heavy smoker: ≥ 30 cigarettes/day	3357 (3.8%)	3563 (4.1%)	3475 (4.0%)	3496 (4.0%)	3170 (3.6%)
*Alcohol intake, N (%)*					
< 1 g/day	11,842 (13.6%)	9684 (11.1%)	8963 (10.3%)	8921 (10.2%)	9550 (11.0%)
1–9.99 g/day	25,849 (29.7%)	26,400 (30.3%)	27,061 (31.1%)	27,666 (31.7%)	30,219 (34.7%)
10–19.99 g/day	15,965 (18.4%)	18,608 (21.4%)	19,360 (22.2%)	20,318 (23.3%)	20,405 (23.4%)
≥ 20 g/day	23,845 (27.4%)	25,549 (29.3%)	25,420 (29.2%)	24,301 (27.9%)	20,830 (23.9%)
None drinkers	9483 (10.9%)	6885 (7.9%)	6342 (7.3%)	5983 (6.9%)	6160 (7.1%)
*Ethnicity, N (%)*					
White	82,375 (93.9%)	82,788 (94.4%)	82,794 (94.4%)	82,664 (94.3%)	82,060 (93.6%)
Mixed Race	463 (0.5%)	485 (0.6%)	525 (0.6%)	514 (0.6%)	629 (0.7%)
Indian/Pakistani	1952 (2.2%)	1424 (1.6%)	1336 (1.5%)	1249 (1.4%)	1124 (1.3%)
Chinese, Asian or other Asian	502 (0.6%)	556 (0.6%)	559 (0.6%)	669 (0.8%)	750 (0.9%)
Black or Black British	1202 (1.4%)	1196 (1.4%)	1284 (1.5%)	1451 (1.7%)	1818 (2.1%)
Other	766 (0.9%)	777 (0.9%)	797 (0.9%)	773 (0.9%)	894 (1.0%)
Diabetes—Yes, N (%)	7068 (8.1%)	4227 (4.8%)	3691 (4.2%)	3431 (3.9%)	3499 (4.0%)
*Women-only covariates:*					
Current HRT users, *N* (%)	7014 (12.8%)	3714 (7.6%)	3060 (6.7%)	2448 (5.6%)	2144 (4.9%)
Current oral contraceptive pill users, *N* (%)	370 (0.7%)	522 (1.1%)	685 (1.5%)	947 (2.2%)	1892 (4.3%)
Menopause status at recruitment, *N* (%)					
Premenopausal	4426 (8.1%)	6975 (14.3%)	8945 (19.7%)	11,533 (26.6%)	16,420 (37.2%)
Postmenopausal	46,494 (84.7%)	37,441 (76.9%)	32,244 (70.9%)	27,605 (63.6%)	23,251 (52.7%)

Values are mean (SD) unless otherwise indicated, percentages include unknown category for missing data

Percentages calculated including missing values and therefore may not add up to 100%

*g* grams, *HRT* hormone replacement therapy, *IGF-I* insulin-like growth factor-I, *N* Number of participants, *Q* quintile, *SD* standard deviation

**Fig. 1:**
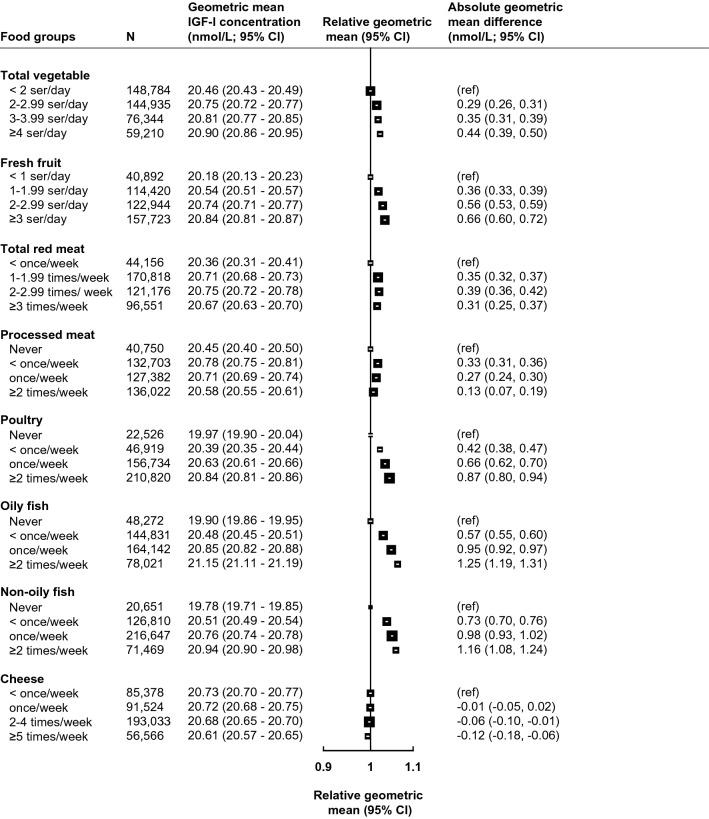
Food groups derived from the recruitment questionnaire in association with geometric mean concentrations of IGF-I (*N* = 438,453). All models are adjusted for sex, age at recruitment, region of recruitment, body mass index, height, physical activity, Townsend deprivation index, education, smoking, alcohol consumption, ethnicity, diabetes, and women-specific covariates: hormone replacement therapy use, oral contraceptive use, and menopausal status. See main text for covariate categories. *CI* confidence intervals, *g* grams, *IGF-I* insulin-like growth factor-I, *N* number of participants, *ref* reference, *ser* servings

Figure 1 presents the multivariable adjusted associations between food group intakes and circulating IGF-I concentrations (see Supplementary Figure S2 for minimally adjusted results), and Table 2 presents absolute and percentage differences in multivariable adjusted geometric mean concentrations of IGF-I between highest and lowest categories of food group intake. The largest magnitudes of associations with IGF-I were observed for oily fish and non-oily fish, where participants who reported consuming these foods ≥ 2 times per week had 1.25 nmol/L (95% confidence interval: 1.19–1.31) and 1.16 nmol/L (1.08–1.24) higher circulating IGF-I concentrations than never consumers, respectively (Fig. 1 and Table 2). Participants who reported consuming poultry ≥ 2 times per week had 0.87 nmol/L (0.80–0.94) higher IGF-I concentrations in comparison to participants who said they never consumed poultry (Fig. 1 and Table 2). For vegetable and fresh fruit intake, small positive associations were observed for individuals in the highest category in comparison with the lowest category, while no associations were observed between intakes of red meat, processed meat, or cheese and circulating IGF-I concentrations (Fig. 1).

**Table 2:** Geometric mean difference and percentage difference of IGF-I concentrations comparing the highest category to the lowest category of food group intake using the baseline IGF-I measurement and in sensitivity analyses using the follow-up IGF-I measurement ~ 4 years after recruitment

Food groups (categories)	Baseline IGF-I measurement (*n* = 438,453)		Follow-up IGF-I measurement (*n* = 16,689)	
	Absolute geometric mean difference (nmol/L; 95% CI)^1^	Percentage geometric mean difference (95% CI)^1^	Absolute geometric mean difference (nmol/L; 95% CI) ^2^	Percentage geometric mean difference (95% CI) ^2^
Total vegetables (≥ 4 ser/day vs. < 2 ser/day)	0.44 (0.39 to 0.50)	2.17% (1.92% to 2.42%)	0.17 (− 0.09 to 0.43)	0.84% (− 0.47% to 2.16%)
Fresh fruit (≥ 3 ser/day vs. < 1 ser/day)	0.66 (0.60 to 0.72)	3.26% (2.97% to 3.55%)	0.31 (0.00 to 0.63)	1.57% (0.00% to 3.15%)
Total red meat (≥ 3 times/week vs. < once/week)	0.31 (0.25 to 0.37)	1.50% (1.21% to 1.80%)	0.26 (− 0.05 to 0.56)	1.29% (− 0.23% to 2.81%)
Processed meat (≥ 2 times/week vs. never)	0.13 (0.07 to 0.19)	0.66% (0.36% to 0.95%)	0.08 (− 0.22 to 0.38)	0.40% (− 1.08% to 1.89%)
Poultry (≥ 2 times/week vs. never)	0.87 (0.80 to 0.94)	4.34% (3.98% to 4.70%)	0.71 (0.38 to 1.05)	3.62% (1.91% to 5.34%)
Oily fish (≥ 2 times/week vs. never)	**1.25 (1.19 to 1.31)**	**6.26% (5.95% to 6.56%)**	**1.04 (0.73 to 1.36)**	**5.35% (3.72% to 6.99%)**
Non-oily fish (≥ 2 times/week vs. never)	**1.16 (1.08 to 1.24)**	**5.85% (5.44% to 6.26%)**	0.92 (0.49 to 1.34)	4.73% (2.54% to 6.91%)
Cheese (≥ 5 times/week vs. < once/week)	− 0.12 (− 0.18 to − 0.06)	− 0.59% (− 0.86% to − 0.31%)	− 0.38 (− 0.67 to − 0.09)	− 1.84% (− 3.27% to − 0.42%)

All models are adjusted for sex, age at recruitment, region of recruitment, body mass index, height, physical activity, Townsend deprivation index, education, smoking, alcohol consumption, ethnicity, diabetes, and women-specific covariates: hormone replacement therapy use, oral contraceptive use, and menopausal status. See main text for covariate categories. Bolded values for food groups indicate a ≥ 5% difference in IGF-I concentrations comparing the highest category to the lowest category

*CI* confidence interval, *IGF-I* insulin-like growth factor-I, *ser* servings

^1^Baseline IGF-I measurement represents difference in highest category of consumption to the lowest category of consumption by food group in Fig. 1

^2^ Follow-up IGF-I measurement represents difference in highest category of consumption to the lowest category of consumption by food group reported at the baseline touchscreen questionnaire (see Supplementary Figure S4)

### Subgroup and sensitivity analyses

In subgroup analyses by sex, the directions of the associations remained the same, although the tests for heterogeneity were statistically significant probably due to differences in the magnitudes of the associations where associations were typically stronger in females (Supplementary Figure S3). In sensitivity analyses restricted to participants with IGF-I measured ~ 4 years after recruitment, associations of IGF-I concentrations ~ 4 years after recruitment with food group intake measured at baseline were slightly weaker, although differences in intakes of oily fish and non-oily fish remained associated with IGF-I concentrations (Table 2 & Supplementary Figure S4).

## Discussion

In this cross-sectional analysis of over 430,000 individuals in the UK Biobank, we found positive associations between intakes of non-oily and oily fish and circulating IGF-I concentrations. We also observed a modest positive association between intake of poultry and IGF-I concentrations. No other strong associations were observed for intakes of fruit, vegetables, red meat, processed meat, or cheese and IGF-I concentrations.

### Fish

Consumption of both oily and non-oily fish was positively associated with circulating IGF-I concentrations, which is consistent with some previous cross-sectional studies assessing intake of fish and IGF-I concentrations [10, 11, 29], although one study did not find an association [9]. Both oily and non-oily fish are good sources of protein, essential amino acids, and minerals, such as zinc and potassium, which have been suggested to be positively associated with IGF-I concentrations [9, 11, 12]. Essential amino acids may up-regulate IGF-I mRNA [30] as well as stimulate pathways in the liver necessary for IGF-I synthesis [31]. Some previous studies have also suggested that intake of polyunsaturated fat, potentially exclusively long chain omega-3 fatty acids [11], may be related with higher IGF-I concentrations [12, 32–34], although the evidence is not consistent [9]. We also observed a relatively strong association for intake of non-oily fish and IGF-I concentrations, which contains less polyunsaturated fat than oily fish. This may suggest that polyunsaturated fatty acids in oily fish may not explain the association between oily fish intake and IGF-I concentrations, and that other compounds present in fish, such as the high protein content, might explain this association.

### Meat

In the current analysis, we also observed that intake of poultry was positively associated with IGF-I concentrations, although this association was weaker than the association with fish intake. To our knowledge, the association between poultry intake and IGF-I concentrations has been null in previous studies [10, 12, 29]. In contrast, intake of red and processed meat was not materially associated with IGF-I concentrations in this study, which is consistent with previous studies [10, 11, 13]. It is unclear whether these different associations with IGF-I between protein-rich foods are attributable to different amino acid compositions or if they might be driven by other components(s) in these foods, such as their mineral content [11, 12]. The amino acid profiles of red meat, poultry, and fish do not differ greatly [35] and therefore may not explain the differences in associations with IGF-I concentrations. However, mineral contents in animal-based foods do differ; for example, fish and poultry may have relatively more magnesium than some red meat [35], and some evidence has suggested that magnesium intake may be positively associated with IGF-I concentrations [10, 12]. Despite this, further research is needed to examine how mineral intake may relate to IGF-I concentrations, and whether minerals have independent associations beyond the intake of protein-rich foods.

### Vegetables and fresh fruit

In this analysis, we observed small positive associations for intakes of both vegetables and fresh fruit with IGF-I concentrations; however, no significant associations were observed in sensitivity analyses using follow-up measurements of IGF-I. Moreover, although these associations were statistically significant in our main analyses, the differences in IGF-I concentrations between lowest and highest categories were small (<3.5%); therefore, these results should be interpreted cautiously and could also be due to associations with other foods. Previous evidence has suggested small inverse or null associations of IGF-I with vegetable intake [11, 12], whereas, for fruit intake, small positive [12, 36] or null[11, 37] associations have been reported and further research is needed.

### Cheese

No association was observed between cheese intake and IGF-I concentration, which is consistent with previous cross-sectional studies [11, 19, 36]. Despite previous studies showing a positive association between intake of dairy products and IGF-I concentrations [9, 11, 19], intake of cheese has not been shown to be associated with IGF-I concentrations [18, 19] suggesting that there may be differences in how dairy products are related with IGF-I concentrations. Moreover, in a subsample of this cohort comprising 11,815 individuals with nutrient intake information, protein from milk and yogurt, but not cheese, was associated with IGF-I concentrations [18]. One possible explanation for the absence of an association for cheese intake is the removal of the whey fraction in cheese production. The whey fraction contains more branched chained amino acids [38], which may be important in stimulating IGF-I production [30]. Frequencies of intakes of dairy products other than cheese were not asked in the recruitment questionnaire in UK Biobank.

This study has some strengths and limitations that should be considered. To the best of our knowledge, this is the largest analysis assessing food group intakes in relation to circulating IGF-I concentrations. We were also able to test the robustness of our results using the follow-up measurement of IGF-I, which was from blood samples collected an average of 4.3 years after recruitment of participants and results were similar. There are also some limitations to consider. Few dietary questions were asked at recruitment, therefore not allowing adjustment for total energy intake and other nutritional factors, as well as limiting the number of foods that could be assessed in relation to IGF-I concentrations in the whole sample. However, we did adjust for BMI, height, and physical activity to try and control for energy intake. As well, we compared differences in IGF-I concentrations between highest and lowest categories of food group, which varied in distribution in the sample and intake amounts between the food groups, thus making it difficult to compare the sizes of the estimates between different foods. There is also measurement error in dietary intakes estimates as only one question was used to determine intakes [26]. As well, we did not adjust for other foods in our analysis due to the limited food groups in the UK Biobank. Due to the observational nature of the study, associations may be subject to unmeasured and residual confounding, and causality cannot be inferred. Moreover, other components of the IGF signalling pathway, such as IGF-II and the IGF binding proteins, which may be important in modulating the effect of IGF-I [1], were not measured in this cohort. The UK Biobank participants are predominantly white and generally healthier than the overall UK population [39]; therefore, the associations might be influenced by selection bias and may not be generalizable to a wider population. Although some associations were observed between food group intakes and circulating IGF-I concentrations, how intakes of these foods relate with IGF-I associated health outcomes, such as cancer risk and bone health, is unclear [40, 41]. Moreover, intakes of these foods may influence health outcomes through other mechanisms external to the IGF-I pathway, and thus further research is needed before conclusions in relation to disease can be made.

In conclusion, we found positive associations between intake of oily fish and non-oily fish and circulating IGF-I concentrations. We also observed a modest positive association between poultry intake and IGF-I concentrations, whereas there were no other strong associations with intakes of vegetables, fruit, red meat, processed meat or cheese. Further research assessing how compounds in these foods, such as individual amino acids and minerals, relate to IGF-I concentrations is warranted. Moreover, studies using methods that may be less susceptible to residual confounding, including large randomised controlled trials using isoenergetic methods, are needed to enhance understanding of how dietary components may modulate IGF-I concentrations and potentially impact health outcomes.

## Supplementary Information

Below is the link to the electronic supplementary material.

Supplementary file1 (DOCX 3934 KB)

## Data Availability

UK Biobank is an open access resource. Bona fide researchers can apply to use the UK Biobank dataset by registering and applying at http://ukbiobank.ac.uk/register-apply/.
