# Supplementary material for: Associations between food group intakes and circulating insulin-like growth factor-I in the UK Biobank: a cross-sectional analysis
Source: Eur J Nutr. Author manuscript; Available in PMC 2023 Feb 7. (PMC9899744; doi:10.1007/s00394-022-02954-4)
Supplement: Supplementary file [file EMS157311-supplement-Supplementary_file.docx]

**Associations between food group intakes and circulating insulin-like growth factor-I in the UK Biobank: a cross sectional analysis**

Cody Z. Watling, Rebecca K. Kelly, Tammy Y. N. Tong, Carmen Piernas, Eleanor L. Watts, Sandar Tin Tin, Anika Knuppel, Julie Schmidt, Ruth C. Travis, Timothy J. Key, Aurora Perez-Cornago

Supplementary Materials

[Supplementary Tables 3](#_Toc109404531)

[Supplementary Table S1. List of medications and UK Biobank codes from baseline used to exclude participants taking medication which may modify insulin-like growth factor-I concentrations 3](#_Toc109404532)

[Supplementary Figures 5](#_Toc109404533)

[Supplemental Figure S1. Flow chart of exclusion criteria and sensitivity analysis restricting to participants who had a follow-up IGF-I measurement. 5](#_Toc109404534)

[Supplementary Figure S2. Minimally-adjusted models for food groups derived from the touchscreen (N=438,453) in association with geometric mean concentrations of IGF-I. 6](#_Toc109404535)

[Supplementary Figure S3. Multivariable-adjusted model for food groups derived from the touchscreen and WebQ 24-hour dietary assessment by sex in association with geometric mean concentrations of IGF-I. 7](#_Toc109404536)

[Supplementary Figure S4. Multivariable-adjusted model for food groups derived from the touchscreen at recruitment in association with geometric mean concentrations of follow-up measurement of IGF-I ~4 years after recruitment (N=16,689). 8](#_Toc109404538)

# Supplementary Tables

## Supplementary Table S1. List of medications and UK Biobank codes from baseline used to exclude participants taking medication which may modify insulin-like growth factor-I concentrations

| Medication code | Medication |
| --- | --- |
| 1140868644 | somatropin |
| 1140857838 | somatrem |
| 1140857840 | somatonorm 4iu injection |
| 1141167490 | somatuline la 30mg injection (pdr)+diluent+syringe |
| 1140857748 | genotropin 12iu multidose injection |
| 1140857750 | somatropin 12iu injection |
| 1140868646 | humatrope(rbe) 4iu(1.3mg) injection (pdr for recon)+diluent |
| 1140868648 | saizen(rmc) 4iu(1.33mg) injection (pdr for recon)+diluent |
| 1140868650 | norditropin(epr) 12iu(4mg) injection (pdr for recon)+diluent |
| 1140884544 | leuprorelin |
| 1141157394 | goserelin product |
| 1140870194 | goserelin |
| 1140870196 | zoladex 3.6 mg implant |
| 1140921100 | triptorelin |
| 1141189852 | decapeptyl sr 3mg injection (pdr for recon)+diluent |
| 1141189772 | gonapeptyl depot 3.75mg inj (pdr for recon)+solv p/f syringe |
| 1140870248 | buserelin |
| 1141157392 | buserelin product |
| 1140870252 | suprefact 100micrograms nasal spray |
| 1140868490 | gestanin 5mg tablet |
| 1140870084 | depostat 200mg/2ml oily injection |
| 1140876638 | cyproterone acetate+ethinyloestradiol |
| 1141192344 | cyproterone acetate+ethinylestradiol |
| 1140884634 | cyproterone |
| 1140868524 | androcur 50mg tablet |
| 1140869270 | medroxyprogesterone |
| 1141190580 | conjugated oestrogens 0.3mg / medroxyprogesterone 1.5mg tab |
| 1140864232 | provera 2.5mg tablet |
| 1140857620 | depo-provera 50mg/1ml injection |
| 1140870274 | flutamide |
| 1140917306 | bicalutamide |
| 1140917310 | casodex 50mg tablet |
| 1141179886 | propecia 1mg tablet |
| 1140928222 | andropatch 2.5mg/24hours transdermal patch |
| 1140910802 | androstanazol |
| 1141193272 | testogel 50mg gel 5g sachet |
| 1141166354 | testoderm 6mg/24hours transdermal patch |
| 1140868534 | primoteston depot 250mg/1ml oily injection |
| 1140868536 | restandol 40mg capsule |
| 1140868538 | sustanon 100 oily injection |
| 1140864502 | testotop tts 15mg transdermal patch |
| 1141167430 | lanreotide |
| 1140870200 | octreotide |
| 1140870208 | sandostatin 50micrograms/1ml injection |
| 1141195128 | pegvisomant |
| 1141195032 | somavert 10mg injection (pdr for recon) +solvent |
| 1140857656 | methyltestosterone product |
| 1140857668 | virormone-oral 5mg tablet |
| 1140857736 | virormone 10mg/1ml injection |
| 1140865136 | yohimbine/pemoline/methyltestosterone |
| 1140868532 | testosterone product |
| 1140868528 | pro-viron 25mg tablet |
| 1140868526 | mesterolone |
| 1140868524 | androcur 50mg tablet |
| 1140868534 | primoteston depot 250mg/1ml oily injection |
| 1140868536 | restandol 40mg capsule |
| 1140868538 | sustanon 100 oily injection |
| 1140868550 | finasteride |
| 1140868608 | proscar 5mg tablet |
| 1140868614 | deca-durabolin 25mg/1ml oily injection |
| 1140868618 | stanozolol |
| 1140868620 | stromba 5mg tablet |
| 1141179886 | propecia 1mg tablet |
| 1141192000 | dutasteride |
| 1141192004 | avodart 500micrograms capsule |
| 1140868968 | danazol |
| 1140870284 | prostap sr 3.75mg injection (pdr for recon)+diluent+kit |
| 1141201718 | nebido 1000mg/4ml solution for injection |
| 1140923018 | anastrozole |
| 1141171100 | exemestane |
| 1141145896 | letrozole |
| 1140870164 | tamoxifen |
| 1140888684 | diazoxide |
| 1140928276 | humatrope(rbe) 18iu(6mg) inj cartridge(pdr for recon)+dil |
| 1141189090 | rosiglitazone 1mg |
| 1140874686 | glucophage 500mg tablet |
| 1141190802 | nutropinaq 10mg(30iu)/2ml injection cartridge |
| 1140923890 | zomacton(rbe) 12iu(4mg) injection (pdr for recon)+diluent |
| 1140868810 | geref 50 injection (pdr for recon)+solvent |
| 1140909918 | biosynthetic human growth hormone |
| 1140882976 | growth hormone product |

# Supplementary Figures

**503,317 participants recruited to UK Biobank**

**64,864 participants excluded:**

• **824** participants withdrew consent

• **27,174** participants diagnosed with cancer (excluding non-melanoma skin cancer)

• **4,077** participants taking medication which may alter IGF-I concentrations

• **32,789** participants without measured insulin-like growth factor-I at recruitment

**438,453 participants eligible for touchscreen analysis**

**Sensitivity analysis: follow-up IGF-I measurement**

**486,628 participants excluded:**

• **824** participants withdrew consent

• **27,174** participants diagnosed with cancer (excluding non-melanoma skin cancer)

• **4,077** participants taking medication which may alter IGF-I concentrations

• **454,553** did not have IGF-I measured a second time

**16,689 in sensitivity analysis**

## Supplemental Figure S1. Flow chart of exclusion criteria and sensitivity analysis restricting to participants who had a follow-up IGF-I measurement.

Abbreviations: IGF-I, insulin-like growth factor-I.

## Supplementary Figure S2. Minimally-adjusted models for food groups derived from the touchscreen (N=438,453) in association with geometric mean concentrations of IGF-I.

All models are adjusted for sex and age at recruitment.

Food groups derived from the touchscreen questionnaire completed by all participants at recruitment.

Abbreviations: CI, confidence intervals; g, grams; IGF-I, insulin-like growth factor-I; N, number of participants, ser, servings.

## Supplementary Figure S3. Multivariable-adjusted model for food groups derived from the touchscreen and WebQ 24-hour dietary assessment by sex in association with geometric mean concentrations of IGF-I.

All models are adjusted for age at recruitment, region of recruitment, body mass index, height, physical activity, Townsend deprivation index, education, smoking, alcohol consumption, ethnicity, diabetes, and women specific covariates: hormone replacement therapy use, oral contraceptive use, and menopausal status.

Food groups derived from the touchscreen questionnaire completed by all participants at recruitment.

Chi-squared and p-values for heterogeneity represent values from likelihood ratio tests for adding an interaction term between sex and food categories and testing for significant model fit.

Abbreviations: het, heterogeneity; IGF-I, insulin-like growth factor-I; N, number of participants, ser, servings.

##

## Supplementary Figure S4. Multivariable-adjusted model for food groups derived from the touchscreen at recruitment in association with geometric mean concentrations of follow-up measurement of IGF-I ~4 years after recruitment (N=16,689).

All models are adjusted for sex, age at recruitment, region of recruitment, body mass index, height, physical activity, Townsend deprivation index, education, smoking, alcohol consumption, ethnicity, diabetes, and women specific covariates: hormone replacement therapy use, oral contraceptive use, and menopausal status.

Food groups derived from the touchscreen questionnaire completed by all participants at recruitment. Participants are excluded who responded ‘prefer not to answer’ or ‘do not know’ for each respective food group therefore not adding up to 16,689.

Participants had IGF-I measured during follow-up. Mean follow-up time for touchscreen analyses 4.3 years.

Abbreviations: CI, confidence intervals; IGF-I, insulin-like growth factor-I; N, number of participants ser, servings.
